# Complete remission in a pretreated, microsatellite-stable, *KRAS*-mutated colon cancer patient after treatment with sintilimab and bevacizumab and platinum-based chemotherapy: a case report and literature review

**DOI:** 10.3389/fimmu.2024.1354613

**Published:** 2024-03-28

**Authors:** Lijuan He, Haiyuan Li, Yunpeng Wang, Weidong Li, Lei Gao, Bo Xu, Jike Hu, Puyi He, Weigao Pu, Guodong Sun, Zhuanfang Wang, Qinying Han, Ben Liu, Hao Chen

**Affiliations:** ^1^ Lanzhou University Second Hospital, Lanzhou, China; ^2^ Department of Surgical Oncology, Lanzhou University Second Hospital, Lanzhou, China; ^3^ Department of Pathology, Lanzhou University Second Hospital, Lanzhou, China; ^4^ Gansu Provincial Key Laboratory Of Environmental Oncology, Lanzhou, China

**Keywords:** colon cancer, MSS, KRAS mutation, immunotherapy, bevacizumab, sintilimab, recurrence, metastasis

## Abstract

Metastatic colon cancer remains an incurable disease, and it is difficult for existing treatments to achieve the desired clinical outcome, especially for colon cancer patients who have received first-line treatment. Although immune checkpoint inhibitors (ICIs) have demonstrated durable clinical efficacy in a variety of solid tumors, their response requires an inflammatory tumor microenvironment. However, microsatellite-stable (*MSS*) colon cancer, which accounts for the majority of colorectal cancers, is a cold tumor that does not respond well to ICIs. Combination regimens open the door to the utility of ICIs in cold tumors. Although combination therapies have shown their advantage even for *MSS* colon cancer, it remains unclear whether combination therapies show their advantage in patients with pretreated metastatic colon cancer. We report a patient who has achieved complete remission and good tolerance with sintilimab plus bevacizumab and platinum-based chemotherapy after postoperative recurrence. The patient had *KRAS* mutation and *MSS*-type colon cancer, and his PD-1^+^CD8^+^ and CD3^−^CD19^−^CD14^+^CD16^−^HLA-DR were both positive. He has achieved a progression-free survival of 43 months and is still being followed up at our center. The above results suggest that this therapeutic regimen is a promising treatment modality for the management of pretreated, *MSS*-type and *KRAS*-mutated metastatic colorectal cancer although its application to the general public still needs to be validated in clinical trials.

## Introduction

Colon cancer (CC) is the third most common type of cancer and the second leading cause of cancer-related deaths worldwide (1). Radical surgery alone or in combination with systemic therapy is the mainstay of curing colorectal cancer, but 30% of CC patients recur after radical surgery (2). Right-sided colon cancer is more likely to have multifocal and poor prognostic recurrence (3). For microsatellite-stable (*MSS*) metastatic colon cancer with *KRAS* mutations, chemotherapy combined with bevacizumab is recommended after surgery or progression. However, 22% of locally recurrent colon cancer re-recurs after surgery (2). No effective therapy exists for recurrent and metastatic colon cancer.

Immunotherapy, especially immune checkpoint inhibitors (ICIs), has demonstrated durable clinical benefit in many solid tumors (4, 5). While inflammatory tumors such as microsatellite instability (MSI) colorectal cancer respond well to ICIs, ICIs have a poor clinical benefit for CC patients with *MSS*. Fortunately, combination therapy has demonstrated the potential to improve the microenvironment to enhance ICI benefit (6–14). An objective response rate of 84% and a disease control rate of 100% were achieved using sintilimab plus bevacizumab, oxaliplatin, and capecitabine for *RAS*-mutant, *MSS*, unresectable metastatic colorectal cancer in a single-arm, phase II clinical trial (15). For CC with *MSS*, combination therapy holds great promise. However, it remains unclear whether this regimen will achieve favorable tumor reduction in patients with pretreated colon cancer.

Herein, we report a case of successful cytoreduction after PD-1 inhibitor plus anti-angiogenic drugs and chemotherapy applied to *MSS* and *KRAS*-mutant colon cancer that progressed after the failure of postoperative first-line adjuvant chemotherapy. Complete remission and manageable side effects were observed in this case. Currently, this is a remarkable report showing both the tolerance and effectiveness of this regimen for pretreated, *MSS* and *KRAS*-mutant colon cancer.

## Case presentation

**Figure 1:**
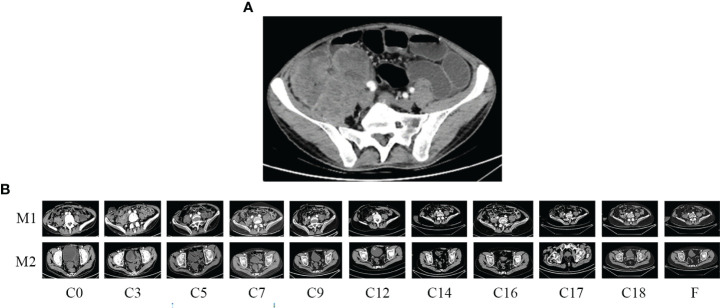
Enhanced computed tomography (CT) scan. **(A)** Before emergent surgery: recurrent and metastatic colon cancer; **(B)** changes in residual lesions throughout the treatment period. M1, para-iliac vascular mass; M2, rectovesical interstitial mass; C, cycle.

**Figure 2:**
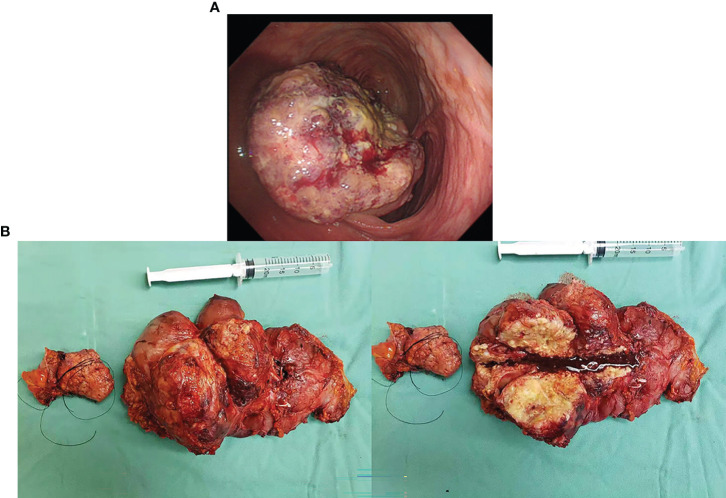
**(A)** Colonoscopy revealed an anastomotic cauliflower-like swelling, that is brittle and bleeds easily on touch. **(B)** The postoperative specimen showed a tumor 20 * 15 cm in size, of exophytic growth, breaking through the stromal layer, and white in cross-section.

A 34-year-old male patient with colon cancer was treated with a right hemicolectomy and three cycles of adjuvant chemotherapy (capecitabine plus cyclophosphamide) at a local hospital. Due to the discovery of metastatic recurrence of colon cancer, he was referred to our hospital in November 2019. This was followed by a series of tests. Clinical examination revealed abdominal distension, right side upper abdominal tenderness, and mass. Serum tumor markers were abnormal: CEA, 58.91 ng/ml (0–3.4 ng/ml), and CA19-9, 228.70 U/ml (0–27 U/ml). Abdominal enhanced computed tomography (CT) showed a giant mass in the hepatic flexure of the colon and multiple abdominal occupations (**Figure 1A**), considered to be recurrence and metastasis of colon cancer. Further colonoscopy revealed a cauliflower-like mass near the transverse colonic anastomosis with a brittle texture, bleeding easily when touched (**Figure 2A**). The patient was then diagnosed as having metastatic and recurrent colon cancer as well as intestinal obstruction, cT4bN1M1b, and multiple metastases in the abdomen, pelvis, and psoas major muscle. Furthermore, the patient’s BMI, 15.04 kg/m^2^; Eastern Cooperative Oncology Group (ECOG), 1 point; Karnofsky Performance Scale (KPS), 80 points; and Nutrition Risk Screening (NRS), 6 points. The patient denies having high blood pressure, diabetes, and other underlying diseases.

### Treatment plan

Members of our multidisciplinary team (MDT) discussed the treatment plan for this case: to remove the obstruction surgically and then provide a postoperative regimen depending on the postoperative pathological findings. After communicating with the patient and family, they agreed to the treatment plan and signed the relevant informed consent, and we, therefore, launched the treatment.

#### Emergent surgery to remove obstruction

**Figure 3:**
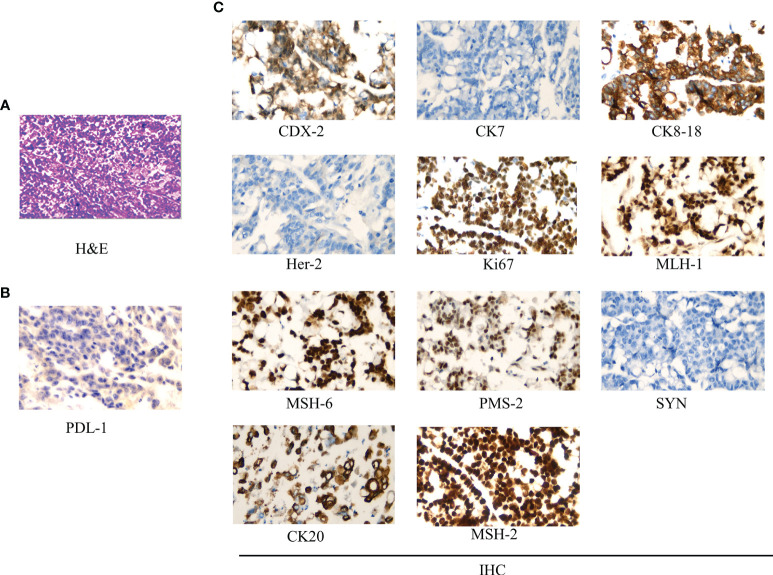
Pathological examination results. **(A)** Hematoxylin–eosin (H&E) staining (*400): low differentiated colonic adenocarcinoma, partly impression cell carcinoma, and partly mucinous adenocarcinoma, accompanied by marked necrosis. **(B)** PDL-1 test results (*400): >50% of tumor cells were PDL-1 positive. **(C)** Immunohistochemistry (IHC) staining (*400): CDX-2, +; CK7, −; CK8/18, +; Her-2 (0); Ki67 positive cells (70%); MLH1, +; MSH6, +; PMS2, +; Syn, −; CK20, +; and MSH2, +.

The surgery relieved the obstruction but failed to remove multiple metastases because of severe adhesion. The postoperative specimen was photographed and revealed a tumor 20 * 15 cm in size, of exophytic growth, breaking through the stromal layer, and white in cross-section (**Figure 2B**). The specimens were sent for pathological examination. H&E staining showed poorly differentiated adenocarcinoma, partly impression cell carcinoma, and partly mucinous adenocarcinoma, accompanied by marked necrosis (**Figure 3A**). Moreover, immunohistochemical (IHC) staining revealed C-erbB-2 (0); CK20, +; CK8/18, +; CK7, −; CDX-2, +; MLH1, +; PMS2, +; MSH2, +; MSH6, +; Syn, −; and Ki67-positive cells (70%) (**Figure 3C**). Postoperative anti-infection, adequate drainage, and nutritional support were given; however, an anastomotic fistula appeared 4 days after surgery. Further jejunostomy was performed resulting in the healing of the anastomotic fistula.

#### Unresectable, metastatic lesions

##### Pretreatment

**Figure 4:**
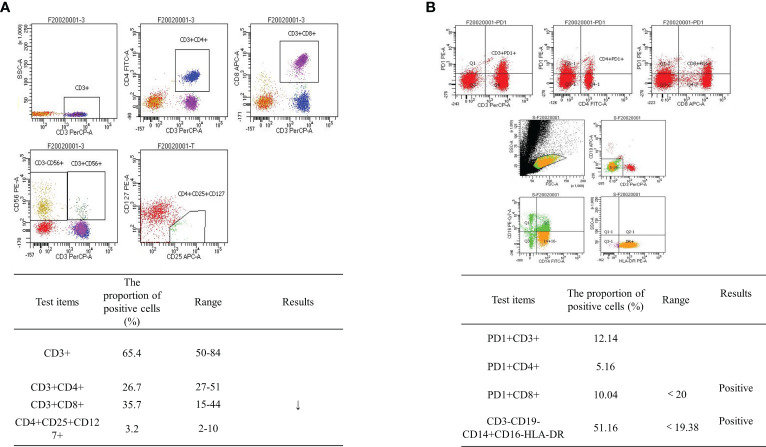
Flow cytometry results. **(A)** Peripheral blood immunization evaluation results: depressed immune function. **(B)** Peripheral blood PD-1 test revealed PD-1^+^CD8^+^-positive and CD3^−^CD19^−^CD14^+^CD16^−^HLA-DR-positive cells.

Abdominal CT was performed after de-obstruction surgery. It showed a right paravalvular iliac mass (**Figure 1B**), a cysto-rectal interstitial mass, and a hydrocele in the right kidney (**Figure 1B**). Genetic testing revealed *KRAS G12D* mutations and *MSS*. Peripheral blood immunization evaluation revealed depressed immune function (**Figure 4A**). The peripheral blood PD-1 test showed that PD-1^+^CD8^+^ and CD3^−^CD19^−^CD14^+^CD16^−^HLA-DR were positive (**Figure 4B**). IHC staining results showed that >50% of tumor cells were PDL-1 positive (**Figure 3B**). Furthermore, the patient's BMI, 16 kg/m^2^; ECOG, 1 point; KPS, 90 points; and NRS, 0 point.

##### Treatment

After the second MDT discussion, it was decided to provide the patient with the following treatment: lobaplatin 50 mg/m^2^, d1, ivgtt; capecitabine 1,250 mg, bid, d1–d14, po; bevacizumab 7.5 mg/kg, d2, ivgtt; and sintilimab 200 mg, d3, ivgtt; 21 days for one cycle, from April 2020 to March 2022 and maintained until September 2022. After a discussion with the patient and his family, they expressed their understanding of the various risks that may occur during treatment, but they were willing to support the doctor’s treatment plan for the sake of the patient’s recovery and signed the informed consent form for the use of the relevant medication.

##### Outcomes

**Figure 5:**
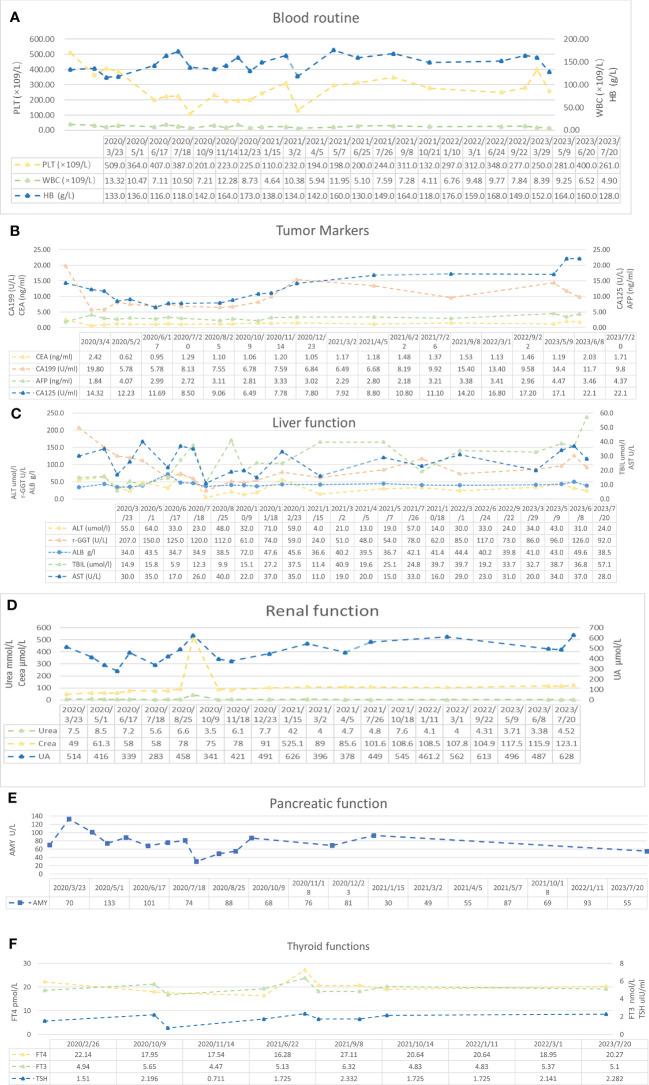
Blood examination results. **(A)** Blood routine results. Reference range: PLT 125–350 * 10^9^/L, HB 130–175 g/L, WBC 3.5–9.5 * 10^9^/L, and RBC 4.3–5.8 * 10^12^/L. **(B)** Tumor marker results. Reference range: AFP 0–7 ng/ml, CEA 0–3.4 ng/ml, CA125 0–35 U/ml, and CA19-9 0–27 U/ml. **(C)** Liver function results. Reference range: ALT 9–50 U/L, AST 15–40 U/L, TBIL <26 μmol/L, γ-GGT 10–60 U/L, and ALB 40–55 g/L. **(D)** Renal function results. Reference range: urea 3.1–8 mmol/L, Crea (creatinine) 57–97 μmol/L, and UA (uric acid) 208–428 μmol/L. **(E)** Pancreatic function results. Reference range: AMY 0–640 U/L. **(F)** Thyroid function results. Reference range: FT4 10.44–24.38 pmol/L, FT3 2.77–6.31 pmol/L, and TSH 0.380–4.340 μIU/ml.

**Figure 6:**
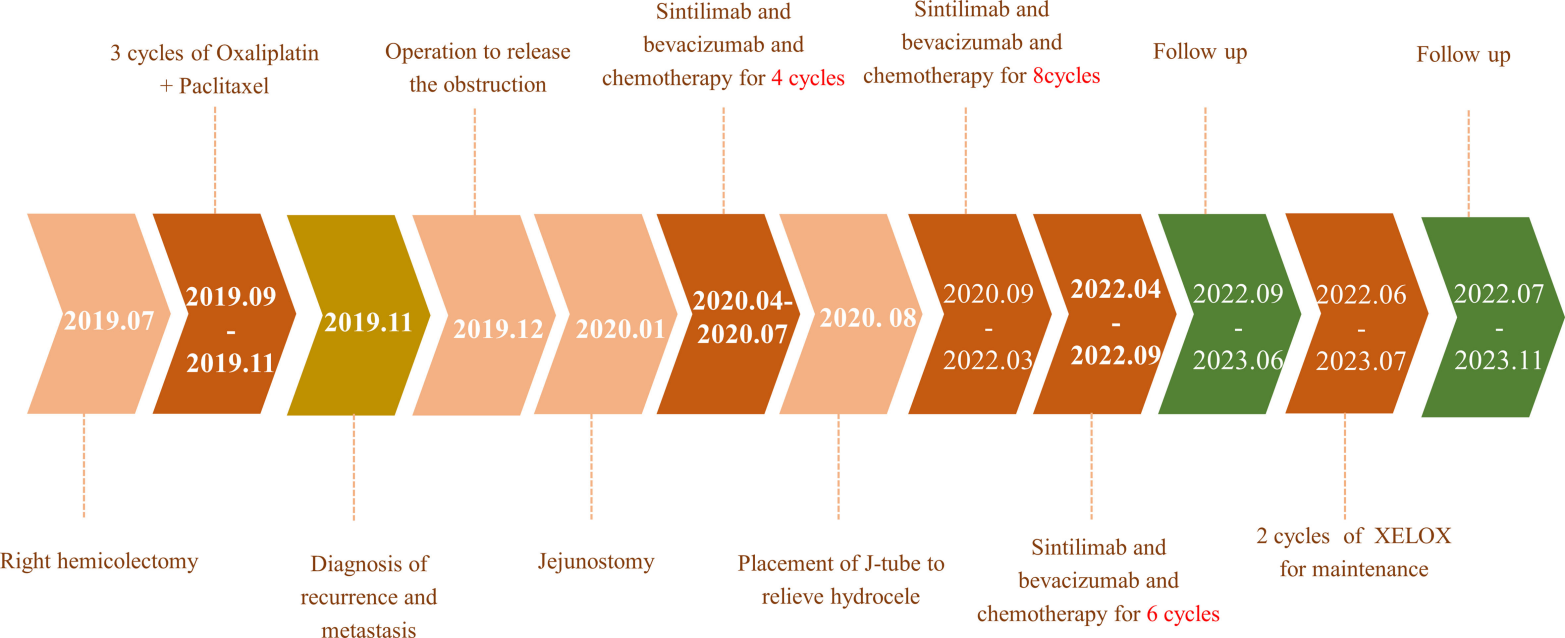
The patient’s timeline.

During the treatment procedure, the patient experienced gastrointestinal side effects such as intermittent nausea and poor appetite. Blood routine results (**Figure 5A**), impaired liver function (**Figure 5C**), and hyperthyroidism (**Figure 5F**) were manageable. Pancreatic function (**Figure 5E**), tumor markers (**Figure 5B**), and abdominal CT (**Figure 1B**) were monitored during the treatment procedure. Regarding the process of disappearance of the lesion, the right paravalvular iliac mass did not shrink in four cycles of medication and resulted in ureteral obstruction, hydronephrosis, and renal hypoplasia (**Figure 5D**). A double J-tube was placed in August 2020 to alleviate the obstruction. After that, target lesions began to decrease in size and vanished completely after 12 cycles of treatment in March 2022. Maintenance medication was given from April 2022 to September 2022, and then the patient was under follow-up. Surveillance from September 2022 to March 2023 showed no abnormalities in imaging or blood analysis. For the early detection of micrometastases, we examined the patient’s CTCs from his peripheral blood in April 2023 and counted 15, and two cycles of maintenance therapy were administered in June and July 2023 with the XELOX regimen. The patient’s timeline is shown in **Figure 6**. His tumor-free survival is 19 months, and his progression-free survival (PFS) is 43 months. The patient is under routine follow-up at our center. The patient regained his appetite and reached a BMI of 21 kg/m^2^.

## Discussion and conclusions

Thirty percent of colon cancer patients develop recurrence after radical surgery (2). Drug resistance and recurrence metastasis remain the two main barriers to effective anticancer therapy for colon cancer, and unsatisfactory and high side effects were detected for existing treatments of this metastatic disease (16). In this case, although the patient experienced delayed progression following right hemicolectomy and three cycles of platinum-based adjuvant chemotherapy, he eventually achieved complete remission and a PFS rate of 43 months and is still being followed up at our center.

Early detection surveillance, personalized treatment, and multidisciplinary cooperation contributed to the complete remission of this patient. Firstly, changes in CTC counts predicted chemotherapy response earlier than imaging serum markers CEA and CA19-9. Furthermore, CTC is the basis of hematogenous metastasis, and monitoring CTC allows the early detection of micrometastasis. In this case, early detection of micrometastases by CTC in the absence of imaging and tumor changes during withdrawal monitoring enabled timely intervention, thereby extending patient survival (17). Secondly, patient-specific, personalized treatment was partly responsible for the complete recovery in this case. Thirdly, patient-centered treatment, timely treatment of the patient’s various complaints, and ensuring that the patient receives antitumor treatment in good condition will yield unexpected results. The patient is striving for survival, and the doctor is actively treating, which allows the patient to achieve complete relief. Last but not least, treating advanced cancer is a comprehensive treatment aimed at prolonging patient survival, and multidisciplinary collaboration is particularly important.

The patient was still tumor-bearing after surgery and even received previous treatment, which added difficulty to the treatment. FOLFOX (5-FU, LV, oxaliplatin) and XELOX are the main therapeutic options for metastatic colon cancer (mCC) (18, 19), while their median PFS is only 8.8 months and 9.3 months, respectively (20). Chemotherapy alone seemed to offer limited improvement in prognosis, and currently, the combination of cytotoxic drugs with targeted agents is recommended for metastatic colon cancer. Mutations in the *RAS* gene are found in 45% of stage IV colorectal cancers (19). These cancers have a poorer prognosis and are resistant to anti-EGFR antibodies (21–24). *MSI* is found in 3% of stage IV colon cancer patients who had a good response to immunotherapy (19, 25). *MSS* accounts for the majority of colorectal cancers. These mutations do not respond well to the immune response. Therefore, the patient described in the case was resistant to both ICIs and cetuximab. Bevacizumab could block VEGF-induced angiogenesis and related immunosuppression (26), exerting a positive impact on ORR, PFS, and OS in patients with mCC (27, 28). In addition, bevacizumab was superior to the right-sided individuals [combined median survival ratio (MSR) = 1.23, 95% CI 1.08 to 1.39] (29). Chemotherapy combined with bevacizumab has been considered as the standard first-line treatment for mCC patients with *MSS* and *RAS* mutation (29, 30). However, 27% of colon cancer patients who used bevacizumab–XELOX relapsed, developed a new colon cancer, or died (31).

**Figure 7:**
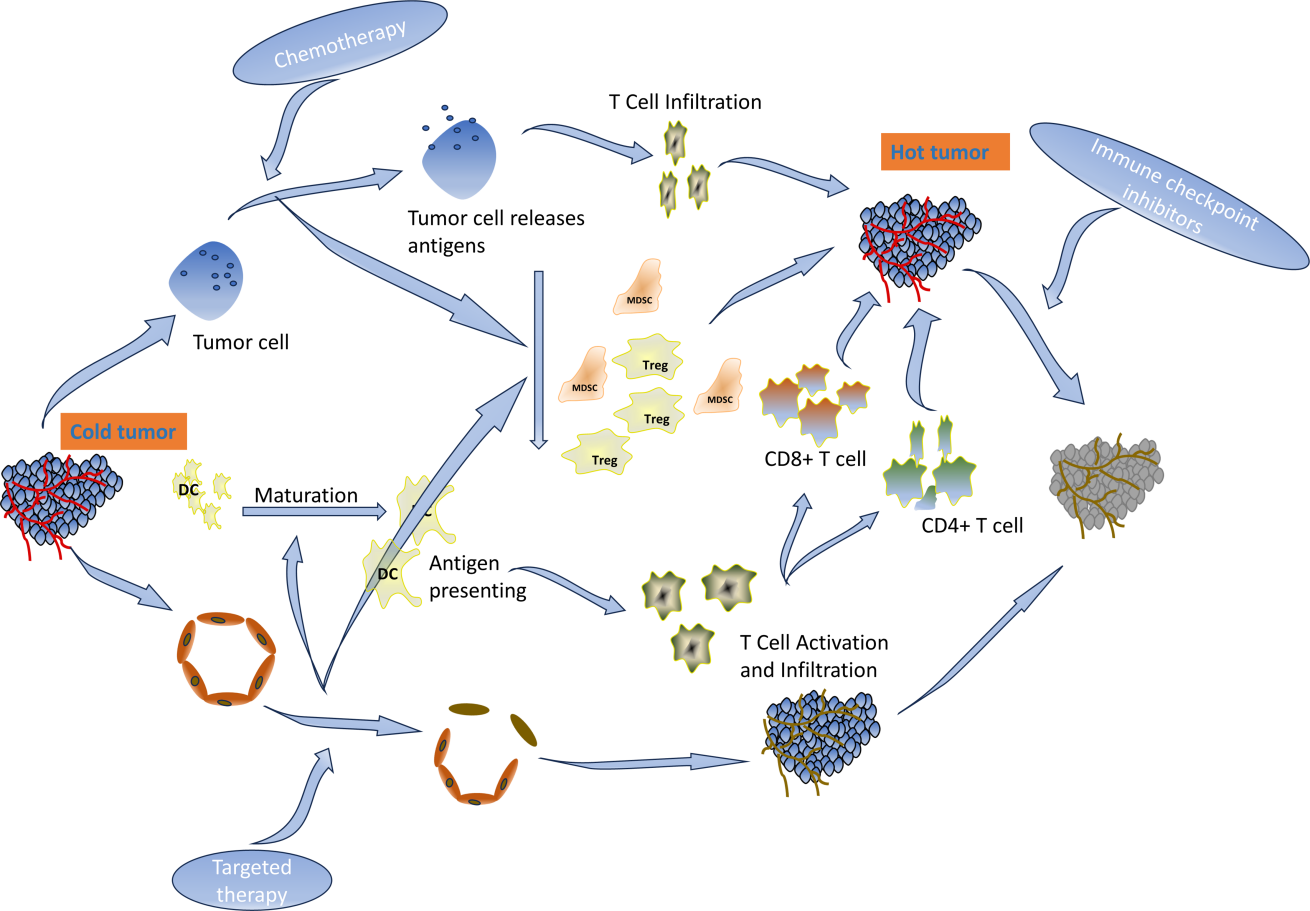
Mechanisms of the patient’s remission.

ICIs have demonstrated durable clinical benefits in many solid tumors (4, 5). The NCCN guidelines recommend pembrolizumab as the standard of care for first-line *MSI-H* advanced colorectal cancer (30). CD8^+^ and PD-1-positive T-cell infiltration was a predictor of response to immunotherapy. The proportion of CD3^−^CD19^−^CD14^+^CD16^−^HLA-DR monocyte subsets in peripheral blood was significantly associated with PD-1 blockade and overall survival (32). However, 95% of mCC cases are *MSS* type and are characterized by the absence or inactivity of CD8 T lymphocytes and reduced expression of checkpoint proteins on the tumor cell. Fortunately, the acquired and natural inflammatory immune microenvironments have the same effect on immunotherapy. Multiple regimens like radiotherapy, kinase inhibitors, immunotherapy (immune activators or other ICIs), chemotherapy, VEGF/EGFR-targeted agents, inhibitors of the PI3K–AKT–mTOR pathway, etc. have been established for combination with ICIs to overcome resistance and enhance ICIs in *MSS* CC (33–37). Chemotherapy drugs destroy tumor cells, release immunogenic antigens, deplete immunosuppressive cells such as Tregs and MDSCs, and enable the expansion of tumor-specific T cells (32, 38). VEGF-targeted therapy can revert the immunosuppressive and angiogenic effects of VEGF (39). ICIs in combination with VEGF-targeted agents and chemotherapy have been shown to be effective in a variety of tumors, including *MSS*-type colon cancer (7, 40). PFS rates of 11.9 months, 13.1 months, and 9.8 months were achieved when different combinations were explored in the CheckMate-9X8 trial (41), AtezoTRIBE trial (42), or NIVACOR trial (43). In addition, first-line treatment with intravenous sintilimab (200 mg, day 1) plus bevacizumab (7.5 mg/kg, day 1), oxaliplatin (135 mg/m^2^, day 1), and oral capecitabine (1 g/m^2^, days 1–14) reported a high ORR and a manageable safety profile (15, 44). Other clinical trials are ongoing (45). Available studies have focused on untreated metastatic colon cancer, and there are currently no relevant clinical trials involving metastatic colon cancer with disease progression after failure of first-line therapy. This case demonstrates that sintilimab in combination with chemotherapy and bevacizumab also improves PFS in previously treated recurrent colon cancer. The function of the regimen is shown in **Figure 7**. After the treatment, there were changes not only in the imaging and hematology results but also in the condition of the patient, who went from a BMI of 15 to 21. The patient regained his appetite and his eyes lit up with hope. In his own words, it was like a nightmare.

Slight adverse effects were observed in the patient in this case after full dosage of the drug, which may be related to younger age, less underlying disease, and drug–drug interactions (**Figure 7**). Furthermore, we chose lobaplatin as the palliative drug because it inhibits colon cancer cells similar to oxaliplatin and only has the specific side effect of platelet inhibition. Perhaps, lopatin rather than oxaliplatin also contributed to the mild adverse effects in this patient, which needs to be further verified.

According to this case report, combining platinum-based chemotherapy with sintilimab and bevacizumab provides a new paradigm for patients with *MSS* and *KRAS*-mutant colon cancer who have received first-line therapy and suffered locoregional recurrence as well as metastasis. However, whether this combination therapy is superior to standard therapy for postoperative recurrent CC remains to be further confirmed through clinical trials due to individual differences. In addition, how to combine therapy rationally and effectively also needs to be further explored.

## Data availability statement

The original contributions presented in the study are included in the article/supplementary material. Further inquiries can be directed to the corresponding author.

## Ethics statement

The patient was treated on a compassionate use basis with the appropriate consent. No other ethical approvals were needed. Consent for administration of this agent was obtained from this patient. In addition, the patient described in this case was aware of, read, and consented to the publication of the final version of the manuscript. The studies were conducted in accordance with the local legislation and institutional requirements. The participants provided their written informed consent to participate in this study. Written informed consent was obtained from the individual(s) for the publication of any potentially identifiable images or data included in this article.

## Author contributions

LH: Writing – review & editing, Writing – original draft, Software, Investigation, Conceptualization. HL: Writing – review & editing, Visualization, Resources, Formal analysis, Conceptualization. YW: Writing – review & editing, Formal analysis, Conceptualization. WL: Writing – review & editing, Visualization. LG: Writing – review & editing, Visualization. BX: Writing – review & editing. JH: Writing – review & editing. PH: Writing – review & editing. WP: Writing – review & editing. GS: Writing – review & editing. ZW: Writing – review & editing. QH: Writing – review & editing. BL: Writing – review & editing. HC: Writing – review & editing, Writing – original draft, Visualization, Resources, Methodology, Funding acquisition, Conceptualization.
